# "Willing to Pay?" Tax Compliance in Britain and Italy: An Experimental Analysis

**DOI:** 10.1371/journal.pone.0150277

**Published:** 2016-02-26

**Authors:** Nan Zhang, Giulia Andrighetto, Stefania Ottone, Ferruccio Ponzano, Sven Steinmo

**Affiliations:** 1 Department of Social and Political Sciences, European University Institute, San Domenico di Fiesole (FI), Italy; 2 Institute of Cognitive Science and Technology, National Research Council, Rome, Italy; 3 Department of Economics, Management and Statistics, University of Milano-Bicocca, Milano, Italy; 4 Department of Law, Politics, Economics and Social Sciences, University of Eastern Piedmont, Alessandria, Italy; 5 Robert Schumann Center for Advanced Studies, European University Institute, San Domenico di Fiesole (FI), Italy; 6 Department of Political Science, University of Colorado, Boulder, United States of America; Universidad de Alicante, ITALY

## Abstract

As shown by the recent crisis, tax evasion poses a significant problem for countries such as Greece, Spain and Italy. While these societies certainly possess weaker fiscal institutions as compared to other EU members, might broader cultural differences between northern and southern Europe also help to explain citizens’ (un)willingness to pay their taxes? To address this question, we conduct laboratory experiments in the UK and Italy, two countries which straddle this North-South divide. Our design allows us to examine citizens’ willingness to contribute to public goods via taxes while holding institutions constant. We report a surprising result: when faced with identical tax institutions, redistribution rules and audit probabilities, Italian participants are significantly more likely to comply than Britons. Overall, our findings cast doubt upon “culturalist” arguments that would attribute cross-country differences in tax compliance to the lack of morality amongst southern European taxpayers.

## Introduction

Modern welfare states face a set of difficult challenges as they adapt to the demographic, economic and political strains of the early 21^st^ century. States must struggle to maintain adequate support for social welfare and educational programs in the face of growing distrust of bureaucratic institutions, intense pressures to cut taxes for politically powerful constituencies, and fiscal burdens arising from an aging population. The ability of governments to collect revenues in an efficient and cost-effective manner is of central importance to how successfully states meet their policy goals. And to ensure a healthy fiscal foundation, states must be able to control (or reduce) tax evasion on the part of their citizens.

Yet, while Western European states generally possess tax systems sharing many of the same formal features [1], actual rates of tax compliance vary widely across these societies [2–7]. Moreover, evasion rates also seem to follow a geographic pattern, with high levels of compliance in northern Europe, and widespread under-reporting in the countries further south. Using the size of the “shadow economy” as a proxy for tax evasion, Schneider and Enste find the lowest compliance rates in Western Europe in Portugal, Spain, Italy and Greece [4].

The literature has advanced several “institutionalist” theories to account for this cross-national variation in tax compliance [8]. In large part, these explanations focus on the relationship between the quality of government and citizens’ willingness to comply with fiscal demands. Specifically, this literature argues that citizens are more likely to pay their taxes if they believe that the government is spending their money honestly and efficiently [3, 7, 9–17]. By contrast, when citizens perceive public institutions as corrupt and wasteful, they are likely to reciprocate by being dishonest in turn [18, 19]. Thus, one explanation for EU-wide differences in levels of tax compliance is that, in southern European countries, people often interact with low quality institutions for which they are (unsurprisingly) unwilling to pay.

A second set of theories links tax compliance to broader cultural norms and values. Within Europe, one important axis of cultural variation concerns how different societies draw the boundaries of moral behavior [18, 20–24]. Specifically, southern European societies are typically characterized as more “familistic” or “collectivist,” and ethical conduct is often assumed to apply to only a small circle of familial or personal relationships, while outside of this circumscribed network, selfish or opportunistic behavior is norm. In the public sphere, individuals follow the rules not out of some internalized sense of “right” and “wrong,” but only when they are coerced to do so [24]. By contrast, northern European societies are often said to emphasize values of “autonomy” or “individualism,” and citizens are presumed to apply the same ethical principles that prevail within familial relations to conduct in the civic realm.

This distinction between what Tabellini has termed “limited” and “generalized” morality has direct implications for the level of tax compliance within a society [24–26]. As many scholars have noted, audit rates and punishment probabilities are insufficient to deter cheating in most cases [10, 27–30], and states must therefore rely upon voluntary compliance to collect fiscal dues. However, if “cheating the system” imposes little moral cost, then the willingness to pay is undermined. Thus, a broader cultural argument would lead us to expect greater tax evasion in southern European countries, *independent of institutional performance*.

Our research attempts to test this hypothesis using cross-cultural behavioral experiments. The advantage of this approach is that it allows us to hold formal institutions (e.g. tax rates, audit probabilities, the efficiency of the state, etc.) constant across countries, and thereby isolate the influence of broader cultural factors on fiscal behavior [9, 31, 32]. In this paper, we report results from two countries—the UK and Italy—which we take as “representatives” of northern and southern Europe. Slemrod estimates the evasion rate in the UK to be around 8% or 9% of GDP [33], while comparative figures for Italy can reach as high as 25% to 30% [34]. Culturally, Italy is often vilified—both in the press as well as in popular opinion—as the quintessential “amoral” society in which people cannot be trusted to behave ethically outside the network of familial and personal relations [20]. By contrast, Britain is rather typical of Protestant northern European societies in terms of cross-national rankings of “autonomy” and “individualism” [21, 22].

Our tax experiment involves over 500 participants across multiple locations in Italy and the UK. The main experimental task consists of a tax compliance scenario in which participants earning real money are asked to report their income under a variety of tax rates and redistributive rules. By comparing income declarations across countries, we are able to investigate whether, independently of the institutions, Italians are indeed less compliant than Britons when faced with identical fiscal choices.

To preview our main results, we find little evidence to support the contention that the morality of tax compliance is weaker in Italy, compared to the UK. Instead, when we average across all of the institutional scenarios in our experiment, we find that the average compliance rate amongst Italian participants is significantly *higher* than amongst the British. As we describe in more detail below, the size of the British-Italian gap in tax compliance varies between each of the different scenarios. However, we emphasize that in every scenario of the experiment, Italian participants report a higher percentage of their income, compared to the British. These results remain robust to the inclusion of a host of demographic controls, and are reproduced in multiple experimental locations in the two countries.

In summary, although stereotypes about the “amorality” of Italian (and, more generally, southern European) taxpayers abound in the popular consciousness, our results suggest that cultural values cannot explain the significant cross-national variation in evasion rates that we observe in the real world. In the concluding section, we discuss several implications of our findings for future work.

## Methods

### Overview

Our experiments were conducted at six universities across the United Kingdom and Italy at various points during the academic year 2013–2014. To respect the anonymity of participants, we do not report the experimental locations in this paper, although details are available from the authors upon request. Our team spent over a year designing and re-designing our experimental protocols to ensure the consistency of the laboratory set-ups and selection pools in each of these locations. Everything from the recruitment methods, to the way participants entered the lab, to the final payments procedure was the same in each session. Our experimental instructions (both oral and on-screen) were also translated and back-translated between Italian and English by different native speakers to ensure consistency in meaning.

Participants were recruited to the experiment using ORSEE [35]. More specifically, each of the universities in which we conducted our study maintains an electronic database of individuals who had expressed interest in participating in behavioral experiments. These participant pools are composed mainly of undergraduate students, but also include a number of non-students and people who had already graduated. Several days prior to the actual sessions, individuals in the database receive an email informing them of the opportunity to take part in an upcoming research project (for a reasonable hourly wage). The email also contained a link where individuals could sign up for one session of their choice from a list of scheduled sessions.

On the day of the experiment, upon arrival in the laboratory, participants were given a randomly-drawn, anonymized ID number and assigned to a corresponding personal computer terminal. Only participants and the researchers were present for each session. The experiments were conducted using zTree [36], participants undertook all experimental tasks via computer, and the terminals were partitioned to ensure that participants could not communicate during the session, nor observe others in the room. Also, to ensure anonymity, we announced that decisions and payments would be linked only to participants’ ID-numbers, and not to individual names.

Once all participants were seated at their individual terminals, we began the session by reading a short introductory script. Participants were informed that they would be asked to complete a number of tasks (which we would gradually describe to them) and make a number of choices. Based on their choices and the choices of the other participants, they would earn experimental currency units (ECUs), which would be converted into local currencies (pounds and euros) at the end of the session. The exchange rate was set so that participants would earn approximately twice the average hourly wage for student employment in the local context. Individuals then took part in the tax compliance experiment, which we describe in the following section. Finally, we collected demographic and attitudinal information via an online survey, which was linked to decisions in the experiment using the anonymized ID numbers. In all, each session of the experiment lasted about 90 minutes, and participants earned an average of 16 euros or 14.5 pounds for their time. All payments were distributed anonymously in cash.

Importantly, prior to beginning the experimental tasks, participants were given no information about the aims of the research project, nor about the decisions we sought to elicit. Also, at no point whatsoever were participants told that they are taking part in a larger study comparing decision-making across national groups. Finally, when moderating each session, we made sure to employ only native speakers (and, in the Italian case, speakers with the “correct” regional accent). These procedures were implemented to ensure that participants would not be subject to national (or group-level) reputational concerns when making their decisions.

### Ethics Statement

Our experiments have been approved by the IRB Committee at the University of Colorado, Boulder, where the principal investigator holds a professorship. Our project has also been approved by the Ethics Council of the European Research Council, and the the European University Institute Ethics committee. Finally, our work has been authorized by all of the Italian and UK laboratories we have used, but we did not undergo a separate university-based IRB review in these cases. All participants signed a written consent form prior to taking part in the study. Participants who wish to leave the experiment early could elect to receive a 5 euro / pound show-up fee. In practice, no individuals refused to participate or dropped out.

### Participants

The data we present in this paper are drawn from 31 different experimental sessions involving a total of 671 participants from a variety of academic disciplines. Because we are interested in comparing specifically British and Italian participants, we retain the data for only native students, whom we define as those individuals born in Britain (Italy) to British (Italian) parents. Because of a misunderstanding at the recruitment stage, one session in [location anonymized] enrolled many immigrants. While we excluded the immigrants’ data, we were also concerned that interacting with a disproportionate number of foreigners may have skewed the behavior of even native-born participants. We therefore drop this session entirely from the analysis. The result leaves us with a subset of 531 participants, of which 281 (52.9%) are from Italy and 250 (47.1%) are from the UK (including the entire sample of 671 participants does not substantively change our results). S1 Table presents descriptive statistics for our participant pools. Overall, 56% of our participants are male, with an average age of 23.8 years (s.d. = 7.7 years).

We note that there are several slight demographic differences between our British and Italian samples. In particular, British participants are significantly more likely to be employed and to report a higher willingness to take risks, while Italians are more likely to study economics. In addition, British participants tend to be more skilled at our real effort task (described below), and hence earn more ECUs in the experiment. On the other hand, we detect no significant differences between the two populations in terms of gender, age, or previous participation in experiments. We control for all of these demographic characteristics in our analyses below.

## Tax Compliance Experiment

### Design and Procedure

The tax compliance experiment proceeds as follows. Participants begin by completing a clerical task, in which they must copy rows of information from a sheet of paper into the computer. For each correctly copied row, participants earn 10 ECUs. Next, participants are asked to declare this income for taxation purposes under different scenarios (an example of the income reporting screen is shown in S1 Fig). In the terminology of the experiment, each scenario constitutes a “round.” Participants are informed that they are free to declare any amount of their income—from 0% to 100%—in each round, with the knowledge that they would pay taxes on only the reported portion of their incomes.

In all, the experiment is composed of nine separate income declaration rounds. Participants have no prior knowledge of the total number of rounds. In each round, we specify different rules for the taxation and redistribution of declared incomes (which we describe shortly). In doing so, we wanted to explore the effects of different causal variables from the tax policy literature. As a consequence, readers will note that our design is similar to previous tax compliance experiments that have also investigated the influence of these variables [27, 37–43]. Importantly, our primary contribution examines how, holding these institutional features constant, tax compliance varies across countries [9, 31].

In Rounds 1 through 3, we implement a flat 30% tax rate, and vary how tax revenues are redistributed to participants, thus simulating behavior under different levels of “efficiency” in providing public goods. In Round 1, there is no redistribution, and all tax revenues are “wasted.” In Round 2, all taxes collected are redistributed on an equal basis to all participants. Finally, in Round 3, all taxes collected are first *doubled* and then redistributed equally to all participants.

By contrast, in Rounds 4 through 6, we hold redistribution constant and instead vary the tax rate. We use a flat tax rate of 10% in Round 4, of 30% in Round 5, and of 50% in Round 6. In each of these rounds, the revenues collected are doubled and redistributed equally to all participants.

Rounds 7 through 9 are more eclectic. In Rounds 7 and 8, we introduce two different progressive taxation schemes. In the first scheme, participants falling within the top 10% of declared incomes pay a 50% tax rate, participants in the bottom 10% of declared incomes pay a 10% tax rate, and everyone else pays a 30% rate. However, participants do not know exactly where they themselves fall in the overall distribution of declared incomes. In the second progressive scheme, all income over 100 ECU is taxed at a 50% rate, income between 50 and 100 ECU is taxed at a 30% rate, and all income below 50 ECU is taxed at a 10% rate. Again, under these two schemes, all taxes collected are doubled and redistributed on an equal basis.

Finally, in Round 9, we donate all tax revenues (collected under a flat 30% tax rate, and then doubled) to a real world charity, rather than redistributing revenues to the group. We selected Oxfam for UK participants, and the UNICEF for Italian participants. The order of experimental scenarios, as well as the rules in each round, are summarized in S2 Table.

In each round, participants were informed that they faced an (independent) 5% probability of being audited, in which case those who have under-reported their income must pay a fine equal to twice the amount of uncollected taxes. Importantly, we reveal the results of any audits only at the conclusion of the experiment. Also, at no point during the experiment do participants have information about whether other participants are audited, nor indeed, whether other participants are honestly declaring their own incomes. In fact, we continuously reminded participants at various points throughout the session that all decisions would be treated anonymously. These procedures were implemented to mitigate the influence of reciprocity, conditional cooperation, reputation or wealth effects.

We also took great care to ensure that the experiment would simulate, as much as possible within a laboratory setting, the private decision problem facing an individual taxpayer. Along these lines, we intentionally incorporate tax language in our protocols, using words such as “income,” “taxes,” and “audit” [9, 44]. While the issue of framing effects in tax experiments is far from settled [27, 45], we believe this design choice offers an improvement over the use of neutrally-framed compliance games in terms of the ability to stimulate taxpayer motivations.

In summary, by comparing how income is reported across our nine taxation and redistribution scenarios, we are able to investigate differences in tax compliance across a range of parameters. Furthermore, because other researchers have also employed similar experimental designs, we are able to use previous studies as an external check on the validity of our results.

### Results and Discussion

**Fig 1:**
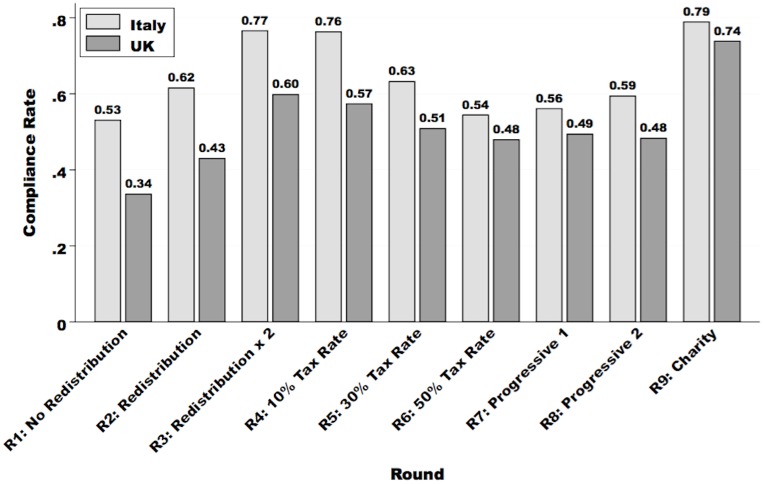
Average compliance rates, by country, in rounds 1 through 9. Bar heights represent the average percentage of earned income that is reported. The compliance rate is lower amongst British participants in every single round. This difference is statistically significant at the 5% level in rounds 1 through 5, and round 8. In addition, it is statistically significant at the 10% level in round 6 (results available from the authors, upon request).

Fig 1 displays the average percentage of earned income that is reported in each of the nine rounds, broken down between British and Italian participants. The vertical axis displays the *average tax compliance rate*, defined as the percentage of total earned income that is declared in each round. Several points stand out from the graph. First, comparing Rounds 1 through 3, we see that compliance responds positively to the efficiency of redistribution: in both countries, individuals are more willing to declare a larger percentage of their income when they know that tax revenues produce more public goods. Secondly, individuals respond to higher tax rates by evading their fiscal obligations: compliance falls as we move from Rounds 4 through 6. These results are in line with previous studies [27, 37–43], and provide us with some assurance about the validity of our experimental design.

Turning now to our main results, we document a surprising cross-national difference in compliance rates: on average, British participants reported a smaller share of their total income in every round as compared to Italians. As shown in S2 Fig, this finding is also fairly consistent across multiple experimental locations in each country.

**Fig 2:**
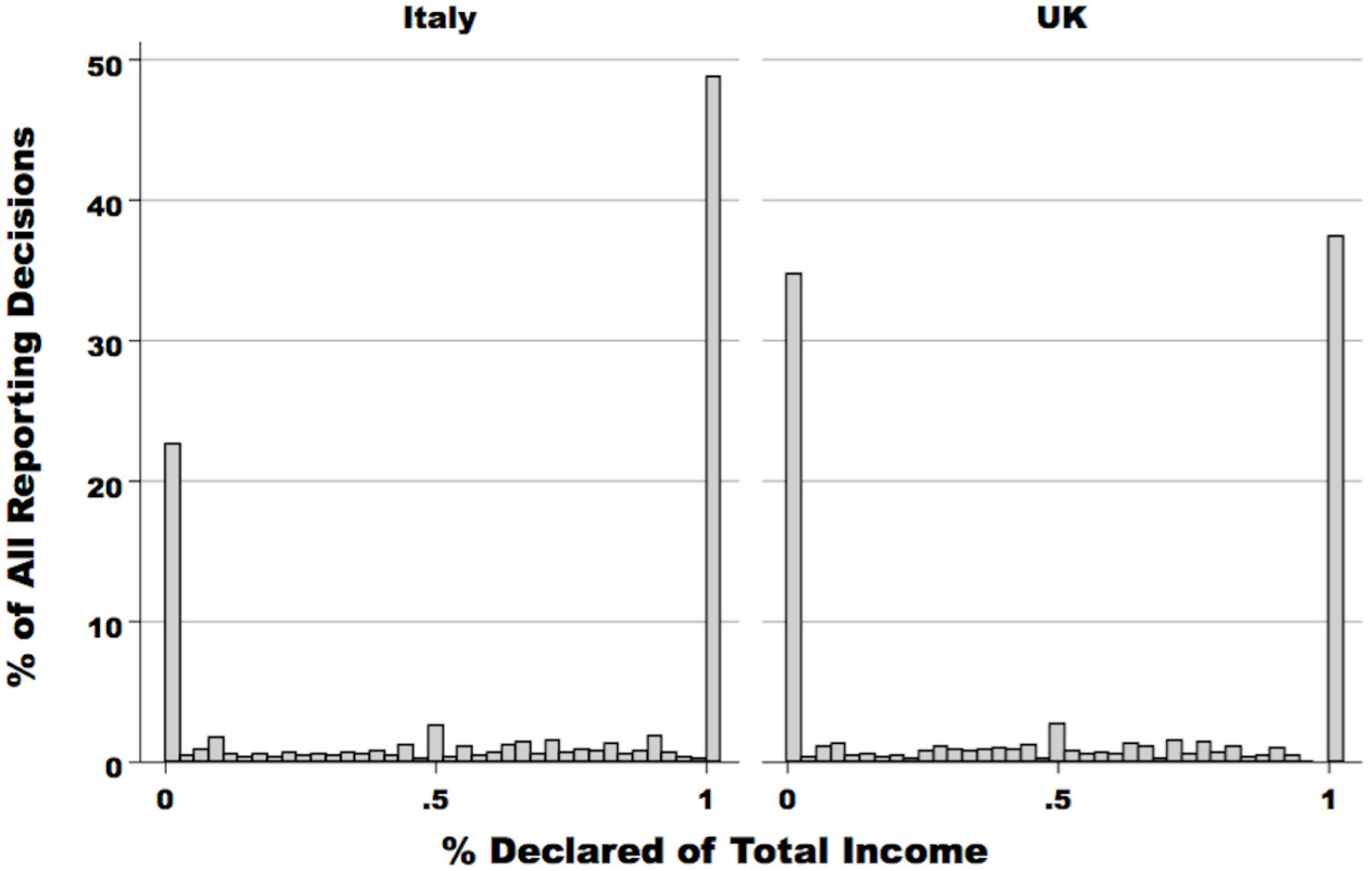
Distribution of compliance decisions, all rounds, by country. Bar heights represent the percentage of reporting decisions in which compliance falls within the ranges [0%–5%], [5%–10%]…[95%–100%]. The distribution is predominately bimodal: in 44.1% of all decisions, participants declare 100% of their earned income, while in 28.4% of all decisions, participants report that they earned 0 income.

While our visual inspection of the the average compliance rate already yields some interesting patterns, such a statistic also hides substantial nuance in participants’ decision-making. Specifically, if we examine the distribution of compliance rates across *all* reporting decisions, we see that the data are not normal (see Fig 2). Rather, the average compliance rate actually aggregates three different outcomes:

1. **Complete Compliance:** In 44.1% of all decisions, participants declare 100% of their earned income.
2. **Partial Evasion:** In 27.5% of all decisions, participants under-report their income to some degree.
3. **Complete Evasion:** In 28.4% of all decisions, participants report that they earned 0 income.

We wish to emphasize that we define these three categories of compliance at the *decision level*, and not at the *individual level*. We adopt this operationalization because individuals’ choices may vary across the different rounds of the experiment (e.g. a participant could declare 0% in round 1, and 100% in round 2), and *a priori*, we had no strong priors about how to classify individuals. For example, if participant A reports 50% of his income in all rounds, while participant B reports 100% of her income in half of the rounds, and 0% in the other half, it was unclear to us that both A and B could be classified as “dishonest” to the same degree. To avoid making (arbitrary) decisions about how to define honesty at the individual level, we opt instead to define categories of compliance at the decision level.

The distributions in Fig 2 are similar to the patterns observed in other experimental studies of tax compliance [27, 46], as well as in “real world” tax declarations. In particular, Alm et al. examined a sample of 1,673 randomly-audited tax returns from the Internal Revenue Service (IRS), focusing solely on self-declared sole-proprietorship income [46]. This income source was selected because it generates no third-party reporting information (e.g. Form W-2 for wage income), and therefore mimics the declaration decision in the laboratory. These authors find that the compliance behavior of real taxpayers also conforms to the U-shaped patterns displayed in Fig 2. In other words, the similarity between our results and Alm et al.’s “taxpayer sample” suggests that our participants are treating the experimental decision-problem as a “real life” problem of tax compliance.

Table 1 examines how this U-shaped pattern differs between the UK and Italy. Columns (1) through (3) of Table 1 display the proportion of decisions in each round characterized by complete evasion. We see that in almost all rounds (except 50% Tax Rate and Charity), a significantly greater percentage of decisions made by British participants result in a declaration of 0. The UK-Italy gaps are substantively large, ranging from about 8% of decisions in Round 7 to almost 18% of decisions in Round 2. In columns (4) through (6), we see the corresponding totals for the proportion of decisions in each round that are completely compliant. Here, the data tell a similar story: in the majority of rounds, significantly more Italians report their entire income. The cross-country gaps range from 10% to 18%. Finally, columns (7) though (9) examine the proportion of partial evasion decisions in each country. Here, however, we see that there are no statistically significant differences. In short, we see that British participants are (a) less likely to report 100% of their incomes, and (b) more likely to report exactly 0 income. By contrast, the proportion of decisions in which individuals engage in partial evasion is almost identical in the two countries.

**Table 1:** Distribution of Complete Evasion, Complete Compliance and Partial Evasion Decisions.

	No. of Decisions: Complete Evasion			No. of Decisions: Complete Compliance			No. of Decisions: Partial Evasion			% of Income Declared by Partial Evaders:		
	(1)	(2)	(3)	(4)	(5)	(6)	(7)	(8)	(9)	(10)	(11)	(12)
	Italy	UK	Diff.	Italy	UK	Diff.	Italy	UK	Diff.	Italy	UK	Diff.
R1: No Redistribuiton	0.33	0.50	-0.17*	0.33	0.19	0.14*	0.34	0.30	0.03	0.59	0.47	0.11*
R2: Redistribution	0.26	0.44	-0.18*	0.48	0.31	0.17*	0.26	0.25	0.01	0.52	0.48	0.03
R3: Redistribution x 2	0.13	0.30	-0.17*	0.66	0.48	0.18*	0.21	0.22	-0.01	0.51	0.55	-0.04
R4: 10% Tax Rate	0.16	0.32	-0.17*	0.64	0.48	0.17*	0.20	0.20	0.00	0.60	0.49	0.11
R5: 30% Tax Rate	0.23	0.34	-0.11*	0.47	0.37	0.10*	0.30	0.29	0.01	0.55	0.49	0.06
R6: 50% Tax Rate	0.31	0.38	-0.07	0.39	0.35	0.04	0.30	0.27	0.03	0.51	0.48	0.03
R7: Progressive 1	0.27	0.35	-0.08*	0.39	0.35	0.04	0.34	0.30	0.04	0.50	0.47	0.02
R8: Progressive 2	0.21	0.32	-0.11*	0.39	0.29	0.10*	0.40	0.39	0.01	0.51	0.49	0.02
R9: Charity	0.14	0.17	-0.03	0.69	0.63	0.06	0.17	0.02	-0.03	0.55	0.53	0.02

N Italy = 281; N UK = 250.

We employed Schlag’s Z-test to test for country-level differences in columns (3), (6) and (9), and Mann-Whitney tests in column (12).

* indicates whether differences between countries are statistically significant at the 5% level.

However, in addition to considering the raw proportion of partial evasions decisions in each round, we must also examine the percentage of taxes declared in this subset. We consider this possibility in columns (10) through (12) of Table 1. Here, we analyze *only* the subset of partial evasion decisions and ask: *conditional* upon engaging in partial evasion, how much income is actually reported? Interestingly, here as well we detect almost no statistically significant differences between Italian and British participants (with the exception of the very first round). In other words, neither the proportion of partial evasion decisions, nor the percentage of income declared in these partial evasion decisions, is very different across our two countries. Rather, it appears that the Italy-UK compliance gap is driven almost entirely by differences in complete compliance and complete evasion.

To what extent can our findings be explained by demographic differences between our participant pools? Controlling for individual-level characteristics that may be correlated with the compliance rate, do Britons still declare less than Italians? To address these questions, we estimate the conditional effect of an Italy country dummy on the compliance rate, holding other factors constant. Following [46], our basic specification is:

$$Y_{i,t}=\beta_{0}+\beta_{1}Italy_{i}+\beta_{2}Income_{i,t}+\beta_{3}X_{i}+\psi_{t}+u_{i}+\epsilon_{i,t}$$

where the dependent variable *Y*_*i*,*t*_ denotes the percentage of income reported by participant *i* in round *t*; *Italy*_*i*_ is a dummy variable for Italian participants; *Income*_*i*,*t*_ is participant *i*’s earned income (standardized) in round *t*; *X*_*i*_ denotes a vector of demographic variables including gender, age, employment status, economics training, previous participation in behavioral experiments, self-reported risk attitudes, and self-reported beliefs about the behavior of other participants; *ψ*_*t*_ is a set of *T* − 1 dummies that capture round fixed effects; *u*_*i*_ are random effects that control for unobservable individual characteristics; and *ϵ*_*i*,*t*_ is an individual-round error term. A more detailed description of these variables is available in the supplementary materials. Following [46], we report results for a (participant) random effects generalized least squares estimation with standard errors corrected for clustering at the individual level. Results are presented in Table 2.

**Table 2:** Estimates of the Compliance Rate.

	(1)	(2)	(3)
Italy	0.13** (0.03)	0.12** (0.03)	0.10** (0.03)
Income (standardized)		-0.03** (0.01)	-0.02 (0.01)
Male			-0.17** (0.03)
Age (standardized)			0.01 (0.01)
Employed			-0.04 (0.03)
Economics Training			-0.09** (0.03)
Previous Participation			-0.09** (0.03)
Risk (standardized)			-0.06** (0.01)
Others Report: “Less”			-0.12** (0.04)
Others Report: “Much Less”			-0.26** (0.04)
Constant	0.52** (0.02)	0.37** (0.02)	0.74** (0.04)
Wald *χ*^2^	20.94**	460.2**	871.8**
Number of Participants	531	531	512
Number of Decisions	4779	4779	4608
Round Fixed Effects	No	Yes	Yes

Panel estimations with participant random effects and clustered (participant level) standard errors. The dependent variable is the percentage of earned income that is declared for tax purposes by individual *i* in round *t*.

** indicates significance at the 1% level.

The number of observations drops slightly once we include demographic covariates in model (3). This is because the experimental tasks were implemented in zTree, while the demographic information was collected separately using Qualtrics survey software. This necessitated that participants enter their anonymous IDs twice: once into zTree, and once again into Qualtrics. Because some participants accidentally entered different IDs into the two systems, we were unable to match their experimental decisions with their demographic data.

Table 2 shows that gender, economics training, past participation in experiments, risk attitudes, and beliefs about others’ behavior are all correlated with compliance. Several results are worth emphasizing: first, unsurprisingly, participants who are more risk-taking also comply less. In addition, British participants self-rate as more risk-taking on average. Thus, the inclusion of the risk variable in the regression with only the *Italy*_*i*_ dummy tends to shrink the cross-country compliance gap from 13% to 8% (results not shown). However, the coefficient on *Italy*_*i*_ is still significant at the 1% level.

Second, individuals who believe that others are dishonest are themselves less likely to comply. This echoes other findings on the importance of social norms for tax compliance [47]. We also note that Italians are on average more pessimistic about the behavior of their counterparts. Thus, controlling for beliefs in the regression slightly increases the size of the cross-country gap from 13% to 14% (results not shown). We also tested whether the relationship between beliefs and compliance differs across our two countries by interacting beliefs with a country dummy (results not shown), but the coefficient on the interaction term was not statistically significant.

Third, in line with earlier studies [48–52], we find that men and economists are less compliant. Since there are slightly more economists amongst Italian participants, controlling for this variable also inflates the cross-country compliance gap from 13% to 15% (results not shown). Fourth, we note that individuals who have participated in experiments in the past report less of their income on average, compared to first-time participants. We believe this result reflects the fact that first-time participants may take the experimental tax frame more seriously, while individuals who participate in multiple experiments may treat the experience as more of a game, and may therefore report less of their income. Finally, we find some evidence that higher earners report less of their income. In model (2) with only the Italy dummy and round fixed effects, a one SD increase in income earned in the clerical task is associated with a 3% drop in the compliance rate (p-value = 0.009). With additional controls (model 3), the association remains negative (2% drop), but is no longer statistically significant at conventional levels (p-value = 0.071).

Most importantly, we find that even controlling for all of these characteristics, the Italian country dummy remains substantively strong and statistically significant. While a simple comparison of the average compliance rate across countries (model 1) shows that Italians report about 13% more of their total income than British participants, this estimate falls only slightly once we include round fixed effects and a control for earned incomes (model 2), as well as other individual level controls (model 3). We also note that our results remain substantively unchanged when the analysis is run using a series of tobit models (available upon request). Importantly, the core finding that Italian participants are systematically more compliant in their fiscal decision-making remains robust in our regression models. In model 3, with the full host of controls, the country gap remains at 10%.

To what extent can we interpret these results as evidence that Italians possess a stronger ethics of tax compliance? Are “culturalist” arguments about the (lack of) morality amongst southern European taxpayers simply wrong? Or might other factors that we have not controlled for account for our surprising results? Here, we discuss two potential confounds: trust in anonymity, and group-level reputational concerns.

First, although we emphasized the anonymity of decisions at multiple points throughout the experiment, the propensity of participants to trust our assurances may vary across countries. For instance, Italian participants may be more suspicious that their anonymity could somehow be compromised, and therefore act more compliantly than they would otherwise.

While we obviously cannot directly control what participants in different societies believe about lab experiments, we have worked to the best of our ability to guarantee to participants that their decisions would be treated anonymously. In particular, information about confidentiality was prominently displayed on the consent forms that participants signed prior to taking part in the experiments. On these forms, it was also stated that participants would be given anonymous ID numbers and randomly assigned to computer stations. Participants also knew that, after the experiment, they would be paid anonymously in sealed envelopes linked only to their ID number. The consent form itself was approved by institutional review boards in two countries, and our confidentiality procedures were scrupulously vetted. We strongly believe that these procedures made it obvious to participants in both Italy and the UK that their anonymity would not be compromised.

A second potential confound relates to a concern we have already noted about maintaining a positive reputation. Specifically, participants in the lab know that they are being observed, and this feeling of being “under the microscope” (especially with respect to a sensitive topic such as tax evasion) may bring about more compliance than would otherwise prevail in the “real world.” Furthermore, the size of this effect may differ between Italy and the UK. In particular, Italians may be more concerned about their collective reputation as a “high evasion” nation. They may therefore act more compliantly in the experiment in order to show their rejection of this stereotype. Importantly, this dynamic may still obtain *even though* (a) our moderators were all native speakers, and (b) participants were not informed that they would be compared to individuals in another country.

While we cannot rule out this alternative explanation for our results, we note that this dynamic is also present in other cross-national experimental studies on tax compliance [9, 31, 53], and does not seem to compromise the validity of earlier findings. Therefore, although we acknowledge that reputation concerns may be potential confounding variable, we believe that these concerns do not play a prominent role in accounting for our results.

## Conclusions and Implications

Turning now to the original question animating our study, what light can our experiment shed on the current fiscal crises facing European countries? Clearly, many northern Europeans (and Americans) hold the view that the difficulties facing Greece, Italy, Spain and others are not simply the product of poor institutions or badly designed public policies, but are instead symptomatic of a far more fundamental problem—one of culture. In a nutshell, southern Europe’s fiscal conundrum is said to stem from the fact that, in such “limited morality” societies, the “moral costs” of non-compliance are insufficient to ensure effective tax collection.

The problem, analytically, is that culture and institutions are difficult to disentangle and are almost certainly interdependent. For example, Italians may readily cheat on their taxes in “real life” precisely *because* they believe that the state—in corruptly and inefficiently using their tax revenue—is also cheating them. In fact, recent public opinion polls have shown that southern Europeans give their governments consistently low scores on control of corruption and the quality of service delivery [54, 55]. By contrast, northern Europeans’ high willingness to pay may be a direct reflection of their belief that taxes go to support important public services which they value and personally consume.

In this article we have tried to analyze the specific question of whether southern Europeans (in this case, Italians) would behave differently than northern Europeans (in this case, the British) when faced with exactly the *same* institutions. We discovered, much to our own surprise, that British participants are more likely to under-declare their incomes in a tax / public goods experiment than Italians. While we do not pretend that our study has isolated all of the cultural variables that may influence tax compliance decision-making, at a minimum, our results cast doubt on the above-mentioned “culturalist” arguments.

We find these results encouraging. If it were indeed the case that culture dominates institutional structure, then the prospects for Europe—and for much of the developing world today—would be grim indeed. Instead, we show that when given the opportunity to contribute to and share in collective goods on an equal institutional playing field, Italians behave no worse than their British counterparts. Thus, we believe that a specific focus on institutional reforms and improving the “Quality of Government” [19] is likely to yield more significant results than the cultural blame game that too often seeps into the policy debate.

## Supporting Information

S1 Fig Example screenshot: Earnings reporting screen. (PNG)

S2 Fig Compliance rate across all locations. (PNG)

S1 Table Summary of participant characteristics: Italy and the UK. (PDF)

S2 Table Summary of tax reporting rounds. (PDF)

S1 Text English Language Experimental Instructions. (PDF)

S2 Text English Language Questionnaire. (PDF)

S3 Text IRB Approval Letters. (PDF)

S4 Text Detailed Description of Selected Covariates. (PDF)
