# Supplementary figures and images for: "Willing to Pay?" Tax Compliance in Britain and Italy: An Experimental Analysis
Source: PLoS One. 2016 Feb 26;11(2):e0150277. doi: 10.1371/journal.pone.0150277 (PMC4769296; doi:10.1371/journal.pone.0150277)

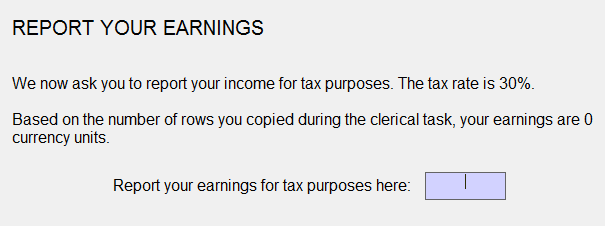

Supplement: S1 Fig — (PNG) [file pone.0150277.s001.png]

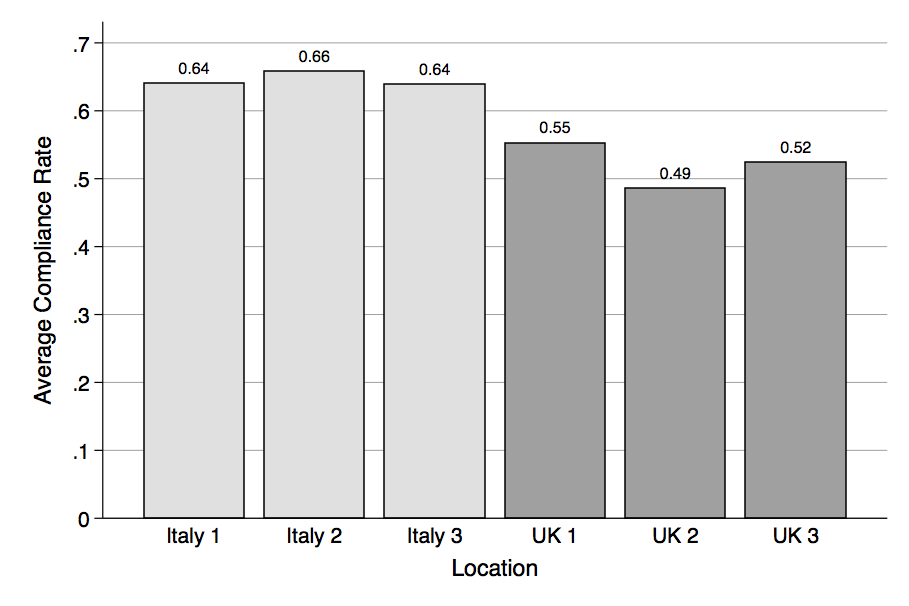

Supplement: S2 Fig — (PNG) [file pone.0150277.s002.png]
