# Supplementary material for: "Willing to Pay?" Tax Compliance in Britain and Italy: An Experimental Analysis
Source: PLoS One. 2016 Feb 26;11(2):e0150277. doi: 10.1371/journal.pone.0150277 (PMC4769296; doi:10.1371/journal.pone.0150277)
Supplement: S1 Table — (PDF) [file pone.0150277.s003.pdf]

Table S1: Summary of participant characteristics: Italy and the UK.

	Obs. (1)	Mean (2)	Std. Dev. (3)	Min. (4)	Max. (5)	Italy Mean (6)	UK Mean (7)	Diff. in Means (8)
Male	527	0.556	0.497	0	1	0.534	0.581	-0.047
Age	527	23.780	7.675	18	73	23.875	23.671	0.205
Employed	526	0.270	0.444	0	1	0.214	0.333	-0.119*
Participated	525	0.821	0.384	0	1	0.817	0.825	-0.008
Economics	527	0.349	0.477	0	1	0.399	0.293	0.106*
Willingness to Take Risks	517	0	1	-2.070	1.886	-0.194	0.215	-0.410*

* indicates whether differences between countries are statistically significant at the 5% level.
We tested for differences in population means using Schlag's Z-test (for dummy variables) and t-tests (for continuous variables).
