# Supplementary material for: "Willing to Pay?" Tax Compliance in Britain and Italy: An Experimental Analysis
Source: PLoS One. 2016 Feb 26;11(2):e0150277. doi: 10.1371/journal.pone.0150277 (PMC4769296; doi:10.1371/journal.pone.0150277)
Supplement: S2 Table — (PDF) [file pone.0150277.s004.pdf]

Table S2: Summary of tax reporting rounds.

**Task**	**Description**
Clerical 1	Earn income that is reported in Rounds 1 through 3
Round 1: No Redistribution	Flat tax rate of 30% on all reported income<br><br>Tax revenues are not redistributed
Round 2: Redistribution	Flat tax rate of 30% on all reported income<br><br>Tax revenues are collected into a common fund, which is redistributed on an equal per capita basis to all participants
Round 3: Redistribution x 2	Flat tax rate of 30% on all reported income<br><br>Tax revenues are collected into a common fund, the amount in the fund is doubled, and then redistributed on an equal per capita basis to all participants
Clerical 2	Earn income that is reported in Rounds 4 through 6
Round 4: 10% Tax Rate	Flat tax rate of 10% on all reported income<br><br>Tax revenues are collected into a common fund, the amount in the fund is doubled, and then redistributed on an equal per capita basis to all participants
Round 5: 30% Tax Rate	Flat tax rate of 30% on all reported income<br><br>Tax revenues are collected into a common fund, the amount in the fund is doubled, and then redistributed on an equal per capita basis to all participants

Continued on next page

Table S2 – *Continued from previous page*

**Task**	**Description**
Round 6: 50% Tax Rate	Flat tax rate of 50% on all reported income<br><br>Tax revenues are collected into a common fund, the amount in the fund is doubled, and then redistributed on an equal per capita basis to all participants
Clerical 3	Earn income that is reported in Rounds 7 through 9
Round 7: Progressive 1	Top 10% of earners in Clerical 3 pay 50% tax on reported income<br><br>Bottom 10% of earners in Clerical 3 pay 10% tax on reported income<br><br>Everyone else pays 30% tax on reported income<br><br>Tax revenues are collected into a common fund, the amount in the fund is doubled, and then redistributed on an equal per capita basis to all participants
Round 8: Progressive 2	participants pay tax of 10% on all reported income under 50 ECU<br><br>participants pay tax of 30% on all reported income between 50 and 100 ECU<br><br>participants pay tax of 50% on all reported income over 100 ECU<br><br>Tax revenues are collected into a common fund, the amount in the fund is doubled, and then redistributed on an equal per capita basis to all participants
Round 9: Charity	Flat tax rate of 30% on all reported income<br><br>Tax revenues are collected into a common fund, the amount in the fund is doubled, and then donated to charity
