# Supplementary material for: "Willing to Pay?" Tax Compliance in Britain and Italy: An Experimental Analysis
Source: PLoS One. 2016 Feb 26;11(2):e0150277. doi: 10.1371/journal.pone.0150277 (PMC4769296; doi:10.1371/journal.pone.0150277)
Supplement: S2 Text — (PDF) [file pone.0150277.s006.pdf]

**Survey Questions:**

| Question | Variable name | Variable type | Answer choices |
|---|---|---|---|
| Age: | age | integer | - R selects age from dropdown menu |
| Gender: | male | integer | Multiple choice radio buttons:<br>0 – Female<br>1 - Male |
| Ethnicity: | ethnic | string | - R types the answer |
| Are you currently employed? | employed | integer | Multiple choice radio buttons:<br>0 – No<br>1 = Yes |
| Are you currently enrolled as a student? | student | integer | Multiple choice radio buttons:<br>0 – No<br>1 = Yes, undergraduate<br>2 = Yes, masters<br>3 = Yes, PhD or other professional degree |
| Major field of study<br>(display only if student != 0) | major | string | - R types the answer |
| Year in your program<br>(display only if student == 1) | progyear | integer | Multiple choice radio buttons:<br>1, 2, 3, 4, 5+ |
| Country of residence: | residence | string | Pulldown list with lab's location as the top entry |
| Country of birth: | birthplace | string | … |
| Mother's country of birth: | m_origin | string | … |
| Father's country of birth: | f_origin | string | … |
| Have you participated in other experiments before? | past_part | integer | Multiple choice radio buttons:<br>0 – No<br>1 = Yes |
| If yes, how many times?<br>(display only if past_part == 1) | times_part | integer | Multiple choice radio buttons:<br>1 = Once<br>2 = Twice<br>3 = 3 -5 times<br>4 = More than 5 times<br>5 = More than 10 times<br>(code as 0 if past_part == 0) |
| How many other participants in today's session do you know by name? | num_friends | integer | Pull down list of numbers from 0 to N |

| | | |
|---|---|---|
| **Listed below are various areas of government spending. Please indicate whether you would like to see more or less government spending in each area. Remember that if you check "much more," it might require a tax increase to pay for it.** | | |
| The environment | Spend_envir | Multiple choice radio buttons:<br>1=Spend much more<br>2=Spend more<br>3=Spend the same as now<br>4=Spend less<br>5=Spend much less |
| Health | Spend_health | … |
| Police and Law enforcement | Spend_police | … |
| education | Spend_educ | … |
| Military | Spend_military | … |
| Old age Pension | Spend_pension | … |
| Unemployment insurance | Spend_unemployment | … |
| Arts and culture | Spend_culture | … |

| | | |
|---|---|---|
| **Now we'd like you to tell us your views on various issues. How would you place your views on this scale? "1" means you agree completely with the statement on the left; "10" means you agree completely with the statement on the right; and if your views fall somewhere in between, you can choose any number in between.** | | |
| Incomes should be made more equal<br><br>We need larger income differences as incentives for individual effort | Equality_vs_incentive | 1 2 3 4 5 6 7 8 9 10 |
| Private ownership of business and industry should be increased<br><br>Government ownership of business and industry should be increased | Private_vs_public_ownership | … |
| Government should take more responsibility to ensure that everyone is provided for<br><br>People should take more responsibility to provide for themselves | Govt_vs_idv_resp | … |

| | | | |
|---|---|---|---|
| Competition is good. It stimulates people to work hard and develop new ideas.<br><br>Competition is harmful. It brings out the worst in people. | Competition_good_bad | | … |
| In the long run, hard work usually brings a better life.<br><br>Hard work doesn’t generally bring success-- it’s more a matter of luck and connections. | Hardwork_vs_luck | | … |
| People can only get rich at the expense of others.<br><br>Wealth can grow so that there’s enough for everyone. | Zero_vs_positive_sum | | … |
| **Please tell me whether you think the following actions can always be justified, never be justified, or something in between. “1” means you think the action can never be justified, and “10” means you think the action can always be justified.** | | | |
| Cheating on taxes if you have the chance | cheat_tax | integer | Row of radio buttons:<br>0 1 2 3 4 5 6 7 8 9 10 |
| Claiming government benefits to which you are not entitled | cheat_benefits | … | … |
| **Generally, how would you describe taxes in your country today? We mean all taxes together, including wage deductions, income taxes, taxes on goods and services, and all the rest.** | | | |
| For those with high incomes, are taxes | Tax_high | | 1= Much too high<br>2= Too high<br>3= About right<br>4= Too low<br>5= Much too low |
| For those with middle incomes, are taxes... | Tax_mid | | … |
| For those with low incomes, are taxes... | Tax_low | | … |
| **The following are different statements made about the [national] tax system. Please indicate how much you agree with each of these statements, where 1 means you completely disagree and 4 means you completely agree.** | | | |

| | | | |
|---|---|---|---|
| The more a person earns, the higher their taxes should be | opinion1 | integer | Multiple choice radio buttons:<br>1 = completely disagree<br>2 = disagree<br>3 = agree<br>4 = completely agree |
| Paying taxes is a fundamental duty of citizenship | opinion2 | … | … |
| Not paying taxes is one of the worst crimes a person can commit because it damages the entire community | opinion3 | … | … |
| It is right not to pay taxes if you think they are unfair | opinion4 | … | … |
| Even if a person considers a tax unfair, they should pay the tax and then protest later | opinion5 | … | … |
| High taxes force individuals to evade taxes in order to keep their businesses afloat | opinion6 | … | … |
| It is justifiable not to pay taxes because the government uses tax money to support people who are lazy | Opinion7 | … | … |
| Most people who cheat on their taxes do so because the tax system is too complicated. | Opinion8 | … | … |
| Most people who cheat on their taxes do so because they feel that politicians are corrupt. | opinion9 | … | … |
| Most people who cheat on their taxes do so because the tax rates are too high. | opinion10 | … | … |
| Most people who cheat on their taxes do so because they feel they will not be caught. | Opinion11 | | |
| It is right to pay taxes because through taxes, we can help the most vulnerable in our society | opinion12 | … | … |
| If the government worked more efficiently, people would be more willing to pay taxes. | opinion13 | … | … |
| Tax money should be spent where taxes are collected | opinion14 | … | … |

| | | | |
|---|---|---|---|
| People would be more willing to pay taxes if they had some control over how tax dollars are spent. | opinion15 | … | … |
| Paying taxes is stressful because even people who try to pay their taxes correctly are afraid of making a mistake and being audited | opinion16 | … | … |
| A person is more willing to pay taxes if he or she believes that other people pay their taxes. | opinion17 | … | … |
| Wealthy people and businesses can usually find legal ways to avoid paying their taxes | opinion18 | … | … |
| Paying taxes is the rational thing to do because our tax dollars fund useful and important services | opinion19 | … | … |
| In political matters, people often talk of "the left" and "the right." How would you place your views on this scale, generally speaking?<br><br>- Left (l) vs. Right (r) | leftright | integer | Row of radio buttons:<br>0 1 2 3 4 5 6 7 8 9 10 |
| Are you generally a person who is completely willing to take risks, or do you normally try to avoid taking them? Indicate your feelings about this issue below, in which 0 means completely unwilling to take risks, and 10 means completely willing to take risks<br><br>- Completely unwilling (l) vs. Completely willing (r) | risk | integer | Row of radio buttons:<br>0 1 2 3 4 5 6 7 8 9 10 |
| Compared to the other participants in this experiment, do you think your earnings are above average, average, or<br>below average? | Think_self_earn | | 1=Above Average<br>2=average<br>3-Below Average |

| | | | |
|---|---|---|---|
| Compared to the other participants in this experiment, do you think your performance on the clerical task was above average, average, or below average? | Think_self_perform | | … |
| Do you think most of the participants in the experiment reported their total earnings, less than their total earnings, or much less than their total earnings for tax purposes? | Think_others_report | | 1=Reported their total earnings<br>2=Reported less than their total earnings<br>3=Reported much less than their total earnings |
| In the experiment, did you report your total earnings, less than your total earnings, or much less than your total earnings for tax purposes? | Self_report | | … |
| Our experiment was created to help us better understand individuals' motivations for tax compliance. Could you please explain your motivations for the decisions you made in today's experiment? | motivations | string | |
| What is your religious affiliation?<br>(show only if religious == 1) | relig_affil | string | - R types response |
| Please rate the importance of religion in your life:<br>(show only if religious == 1) | relig_level | integer | Multiple choice radio buttons:<br>1 – very important<br>2 – fairly important<br>3 – not very important |
| How frequently do you attend religious services?<br>(show only if religious == 1) | relig_freq | integer | Multiple choice radio buttons:<br>1 = daily<br>2 = multiple times per week<br>3 = about once a week<br>4 = about once a month<br>5 = only once every few months |
