# Supplementary material for: "Willing to Pay?" Tax Compliance in Britain and Italy: An Experimental Analysis
Source: PLoS One. 2016 Feb 26;11(2):e0150277. doi: 10.1371/journal.pone.0150277 (PMC4769296; doi:10.1371/journal.pone.0150277)
Supplement: S3 Text — (PDF) [file pone.0150277.s007.pdf]

University of Colorado Boulder

Institutional Review Board
563 UCB
Boulder, CO 80309
Phone: 303.735.3702
Fax: 303.735.5185
FWA: 00003492

24-Sep-2013

Initial Approval - Expedited

Steinmo, Sven
Protocol #: 13-0404
Title: Willing To Pay?

Dear Sven Steinmo,

The Institutional Review Board (IRB) has approved this protocol in accordance with Federal Regulations at 45 CFR 46.

Initial Approval Date: 24-Sep-2013
Expiration Date: 23-Sep-2014
Documents Approved: Survey questions; recruitment scripts; Flyer; Post-research email; 13-0404 Consent Form (24Sep13); 13-0404 Protocol (24Sep13);
Documents Reviewed: Consent form; SVO question; Protocol; HRP-211: FORM - Initial Application;
Review Cycle: 12 months
Expedited Category: 7

Click here to find the approved documents for this protocol: Approved Documents

Regulations require that this protocol be renewed prior to the above expiration date. The IRB will provide a reminder prior to the expiration date, but it is your responsibility to ensure that the continuing review form is received in sufficient time to be reviewed prior to the expiration date.

Changes to your protocol must be submitted to the IRB for review and approval prior to their implementation. This includes changes to the consent form, principal investigator, protocol, etc.

All events that meet reporting criteria must be submitted within 5 business days from notification of the event. Any study-related death must be reported immediately (within 24 hours) upon learning of the death.

The IRB has approved this protocol in accordance with federal regulations, university policies and ethical standards for the protection of human subjects. In accordance with federal regulation at 45 CFR 46.112, research that has been approved by the IRB may be subject to further appropriate review and approval or disapproval by officials of the institution. The investigator is responsible for knowing and complying with all applicable research regulations and policies including, but not limited to, Environmental Health and Safety, Scientific Advisory and Review Committee, Clinical and Translational Research Center, and Wardenburg Health Center and Pharmacy policies. Approval by the IRB does not imply approval by any other entity.

Please contact the IRB office at 303-735-3702 if you have any questions about this letter or about IRB procedures.

Douglas Grafel
IRB Admin Review Coordinator
Institutional Review Board
