# Supplementary material for: "Willing to Pay?" Tax Compliance in Britain and Italy: An Experimental Analysis
Source: PLoS One. 2016 Feb 26;11(2):e0150277. doi: 10.1371/journal.pone.0150277 (PMC4769296; doi:10.1371/journal.pone.0150277)
Supplement: S4 Text — (PDF) [file pone.0150277.s008.pdf]

Detailed Description of Selected Covariates

Risk attitudes. We measure risk-attitudes with a survey item which asks participants to rank themselves on a 10-point scale, with 1 signifying a person who "normally tries to avoid taking risks" and 10 signifying someone who is "completely willing to take risks." The question is very similar to one asked on the German Socio-Economic Panel (SOEP). While simple to understand, this method presents a drawback in that individuals' answers are not incentivized. While we appreciate this concern, we would point to two counterarguments supporting the validity of our measure. First, even though risk attitudes elicited in this manner might constitute a form of "cheap talk," there is no reason to suspect that individuals would intentionally misrepresent their risk preferences: nothing can be gained (either in terms of money, reputation or self-image) from expressing a "low" or "high" appetite for risk. Secondly, Dohmen et al. conducted a "horse race" between different risk elicitation methods (both incentivized and non-incentivized) in terms of the ability to explain behaviors such as holding stocks, occupational choices, and smoking, and find that the general risk question "generates the best all-round predictor of risk behavior" [1]. In the end, we agree with Charness et al. that the elicitation approach should be tailored to the underlying question to which the research is addressed [2]. Since our aim is solely to measure differences across individuals in risk preferences (as opposed to, for example, estimating specific parameters of a mathematical model), we opt for the simplest method. We believe that the gains from having an easily understandable question outweigh the risks of "cheap talk."

Beliefs about others' behavior. We measure beliefs by simply asking participants: "Do you think most of the participants in the experiment reported (a) their total earnings, (b) less than their total earnings, or (c) much less than their total earnings for tax purposes?" While recognizing the arguments in favor of more complicated and incentivized belief elicitation methods [3], we opted again for a simple survey measure in order to minimize comprehension issues.

Earnings in the real effort task. This unstandardized variable is defined by the number of rows correctly copied in the clerical task. In general, Italians performed worse on the clerical task than British participants (Italy mean = 34.5, s.d. = 10.7; UK mean = 38.4, s.d. = 10.6). The total number of rows of information correctly copied into the computer in each of the different locations is: Italy1 mean = 35.0, s.d. = 8.3; Italy2 mean = 35.7, s.d. = 14.8; Italy3 mean = 32.5, s.d. = 6.8; UK1 mean = 37.6, s.d. = 6.9; UK2 mean = 37.4, s.d. = 8.1; UK3 mean = 37.4, s.d. = 8.3.

References

[1] Dohmen T, Falk A, Huffman D, Sunde U, Schupp J, Wagner G. Individual Risk Attitudes: Measurement, Determinants, and Behavioral Consequences. Journal of the European Economic Association. 2011;9(3):522–550.

[2] Charness G, Gneezy U, Imas A. Experimental Methods: Eliciting Risk Preferences. Journal of Economic Behavior & Organization. 2013;87:43–51

[3] Bicchieri C, Xiao E. Do the Right Thing: But Only if Others Do So. Journal of Behavioral Decision Making. 2009;22(2):191–208.
